# Nonalcoholic Steatohepatitis: A Search for Factual Animal Models

**DOI:** 10.1155/2015/574832

**Published:** 2015-05-03

**Authors:** Sheila Cristina L. Sanches, Leandra Naira Z. Ramalho, Marlei Josiele Augusto, Deisy Mara da Silva, Fernando Silva Ramalho

**Affiliations:** Department of Pathology, School of Medicine of Ribeirão Preto, University of São Paulo, Avenida Bandeirantes 3900, Monte Alegre, 14049-900 Ribeirão Preto, SP, Brazil

## Abstract

Nonalcoholic fatty liver disease (NAFLD) is characterized by hepatic steatosis, which occurs in the absence of alcohol abuse. NAFLD can evolve into progressive liver injury and fibrosis in the form of nonalcoholic steatohepatitis (NASH). Several animal models have been developed to attempt to represent the morphological, biochemical, and clinical features of human NASH. The actual review presents a critical analysis of the most commonly used experimental models of NAFLD/NASH development. These models can be classified into genetic, nutritional, and a combination of genetic and nutritional factors. The main genetic models are *ob/ob* and *db/db* mutant mice and Zucker rats. The principal nutritional models employ methionine- and choline-deficient, high-fat, high-cholesterol and high-cholate, cafeteria, and high-fructose diets. Currently, associations between high-fructose and various compositions of high-fat diets have been widely studied. Previous studies have encountered significant difficulties in developing animal models capable of reproducing human NASH. Some models produce consistent morphological findings, but the induction method differs significantly compared with the pathophysiology of human NASH. Other models precisely represent the clinical and etiological contexts of this disease but fail to provide accurate histopathological representations mainly in the progression from steatosis to liver fibrosis.

## 1. Introduction

Nonalcoholic fatty liver disease (NAFLD) is characterized by abnormal lipid accumulation in hepatocytes, which is known as steatosis and occurs in the absence of alcohol abuse. NAFLD is one of the most common forms of liver disease reported in current clinical practice. The prevalence of NAFLD is estimated to reach 30% of adults in developed countries, and greater than 10% of cases evolve into progressive liver injury in the form of nonalcoholic steatohepatitis (NASH) [1]. Because of the high prevalence of NAFLD in the obese population, NAFLD can be considered one of the manifestations of metabolic syndrome including central obesity, type II diabetes mellitus, hypertension, hyperglycemia, and hyperlipidemia [2]. Some factors also contribute directly to the development of NAFLD, such as a sedentary lifestyle and increased consumption of high-fat foods and beverages with high concentrations of fructose [3].

**Figure 1:**
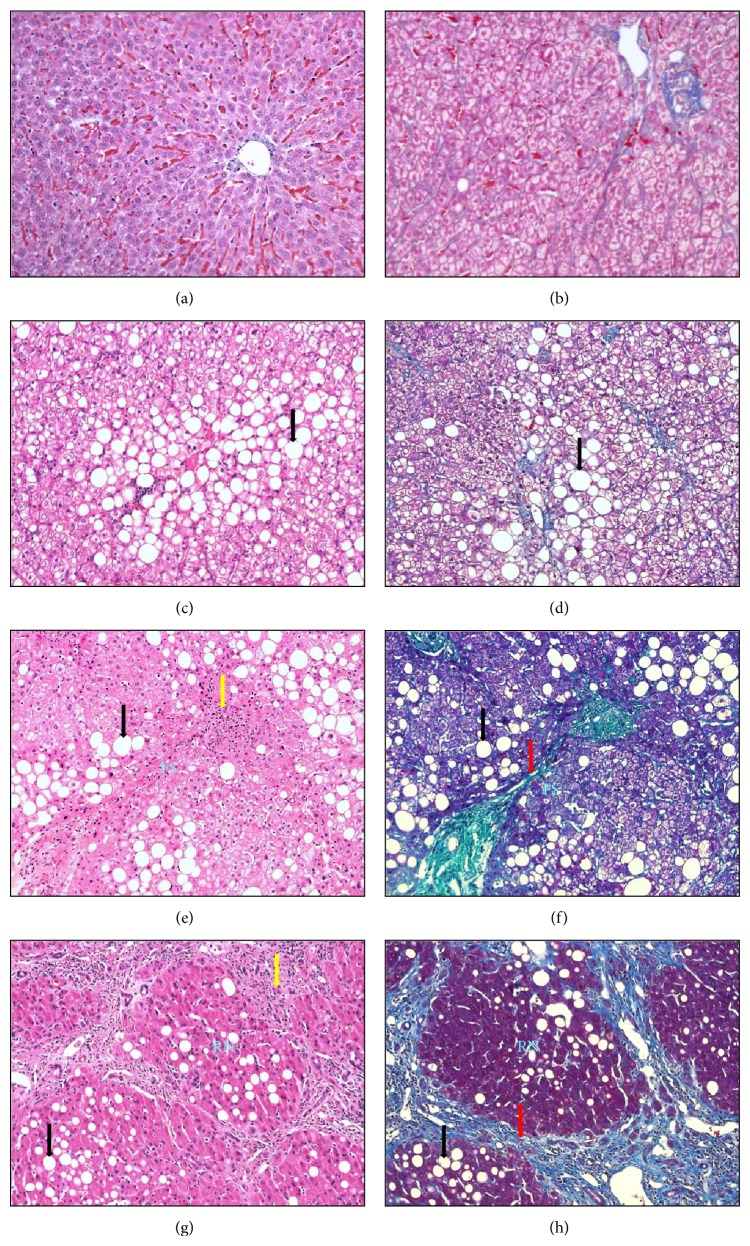
The representative photomicrographs present the progression of the histopathological alterations found in the spectrum of nonalcoholic fatty liver disease (NAFLD): (a)/(b) normal liver: no evidence of steatosis, inflammation, or fibrosis; (c)/(d) liver steatosis: moderate steatosis (34–66%), no evidence of inflammation or fibrosis; (d)/(e) nonalcoholic steatohepatitis (NASH): moderate steatosis (34–66%), mild inflammation, and moderate fibrosis with a fibrous septa (FS); (f)/(g) liver cirrhosis secondary to NASH: moderate steatosis (34–66%), moderate inflammation, and severe fibrosis with a regenerative nodule (RN) (Hematoxylin & Eosin and Masson Trichrome staining, ×200). The black, red, and yellow arrows show steatosis, fibrosis, and inflammation, respectively.

In addition, patients with steatosis, ballooning degeneration, Mallory's hyaline corpuscles, and fibrosis display a higher probability of developing cirrhosis compared with patients exhibiting steatosis alone. Thus, NAFLD includes a wide spectrum of liver abnormalities that may vary from simple steatosis to NASH (Figure 1). In this context, NASH can be identified by the presence of significant fibrosis and necroinflammatory activity in which collagen deposition is manifested as a peculiar perivenular and/or pericellular pattern [4]. Although the majority of patients can be effectively diagnosed with NAFLD using noninvasive tests, liver biopsy remains the gold standard for the accurate assessment of the graduation of steatosis, necroinflammatory changes, and fibrosis and allows NASH and steatosis to be distinguished [5, 6].

A recently developed grading to NASH incorporates the severity of hepatic steatosis, portal and lobular inflammation, and collagen deposition. Like this, the grading of the severity of hepatic steatosis varies as follows: grade 0, minimal or no evidence of steatosis (<5% of hepatocytes affected); grade 1, mild steatosis (5 to 32% of hepatocytes affected); grade 2, moderate to severe steatosis (33 to 66% of hepatocytes affected); grade 3, severe steatosis (>66% of hepatocytes affected). The portal and lobular inflammation is also scored as follows: grade 0, minimal or no evidence of inflammation; grade 1, mild inflammation; grade 2, moderate to severe inflammation; grade 3, severe inflammation. The collagen deposition varies as follows: grade 0, minimal or no evidence of fibrosis; grade 1, mild fibrosis; grade 2, moderate to severe fibrosis; grade 3, severe fibrosis [4]. Another similar method for NASH grading is the SAF system, which comprises a semiquantitative score of steatosis (S), inflammatory activity (A), and fibrosis (F) [7].

The mechanisms involving the pathogenesis of NASH are not completely clarified. One of the main hypotheses is “the theory of the two hits.” According to this theory, the “first hit” for NASH establishment is lipid accumulation in hepatocytes, mostly in the form of triglycerides, which results from an imbalance between the metabolic pathways that promote the hepatocyte uptake and synthesis of fatty acids and those that promote oxidation and export of fatty acids. Insulin resistance, found in obesity and type II diabetes, has been considered the most important factor in the development of hepatic steatosis (the “first hit”). Insulin resistance causes peripheral lipolysis and hyperinsulinemia. Lipolysis increases circulating free fatty acids (FFA) and hepatocyte uptake of fatty acids. Hyperinsulinemia intensifies the hepatic synthesis of fatty acids by inducing glycolysis and favors the accumulation of triglycerides within hepatocytes by decreasing the liver ability to reesterify and export triglycerides [8, 9].

**Figure 2:**
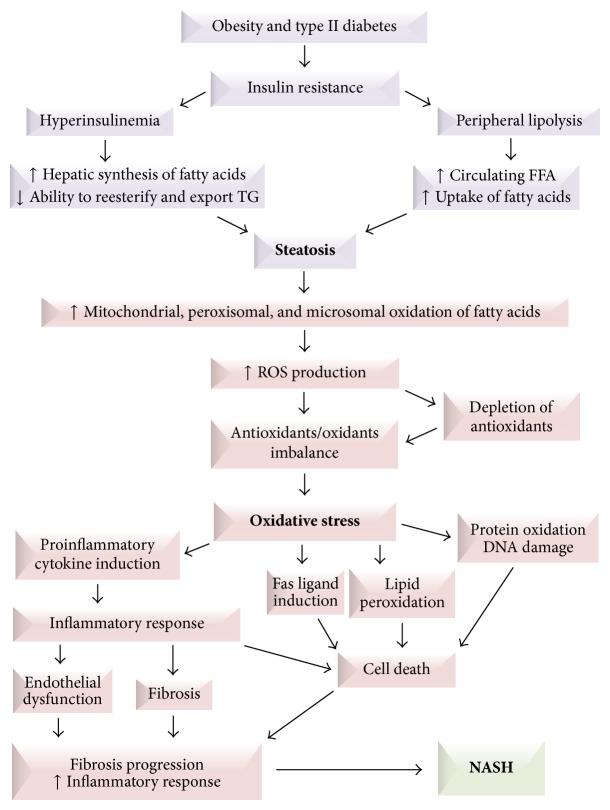
The main mechanisms involved in the pathophysiology of the nonalcoholic steatohepatitis (NASH). FFA: free fatty acids; ROS: reactive oxygen species; TG: triglycerides (adapted from Angulo, 2002 [8]).

In consequence of hepatic steatosis, hepatocytes develop vulnerability to oxidative stress, which may, in large part, be responsible for the progression of NAFLD from simple steatosis to steatosis associated with necroinflammatory activity and fibrosis. Therefore, oxidative stress has been reported as the “second hit.” Mitochondria play a central role in oxidation of fatty acids. Since mitochondrial fatty acid oxidation results in production of free radicals, mitochondria are the major cellular source of reactive oxygen species (ROS), mainly in the form of hydrogen peroxide. Oxidative stress has been described as a disturbance in the equilibrium status of ROS generation and the cellular antioxidant defense system. In hepatic steatosis, the imbalance between scarce endogenous antioxidant reserves and augmented mitochondrial production of free radicals results in oxidative damage to lipids, proteins, and DNA with subsequent cell death [9, 10]. Oxidative stress may trigger steatohepatitis by three main mechanisms: lipid peroxidation, cytokine induction, and induction of Fas ligand. In addition to directly inducing cellular destruction by massive membrane lipoperoxidation, ROS can act as second messengers in the regulation of genes encoding proinflammatory and profibrogenic cytokines such as tumor necrosis factor alpha (TNF-*α*), transforming growth factor beta (TGF-*β*), and interleukin- (IL-) 8. ROS can also induce expression of the Fas ligand in hepatocytes, which normally express the membrane receptor Fas. The Fas ligand on one hepatocyte can interact with Fas on another hepatocyte resulting in fractional liver cell killing (Figure 2) [8, 9].

Multiple intrinsic mechanisms have been suggested to trigger cell death and progression to NASH. However, diverse evidences show that hepatocellular apoptosis is increased both in animal models [11–13] and in human NASH [14–16]. Therefore, apoptosis has been considered the major mechanism of cell death in the NASH context, stimulating liver inflammation and fibrosis. The steatosis-induced oxidative stress promotes cell death through the activation of stress-related signaling pathways such as c-Jun N-terminal kinase (JNK) or p38 mitogen-activated protein (MAP) kinase. Both JNK and p38 MAP kinase are involved in mechanisms of apoptosis [17, 18]. Moreover, increase of the apoptosis frequency may be considered a profibrogenic event in progression of chronic liver diseases. Indeed, apoptosis fragments and ROS can stimulate Kupffer cells to release proinflammatory and profibrogenic cytokines, which induce activation of hepatic stellate cells. These cells are the major producers of extracellular matrix in the tissue repair reactions in response to chronic hepatic injury [19].

The mechanisms that involve apoptosis in NASH were investigated in rats using a high-fat diet for 12 weeks. The results showed that the index of hepatocellular apoptosis was significantly higher in rats fed with high-fat diet. The authors concluded that the hepatocellular apoptosis in this model was promoted by interacting between cyclooxygenase-2 and proinflammatory cytokines (TNF-*α* and IL-6) [20]. A recent study showed the contribution of caspase 3 on liver injury and fibrogenesis and supported a prominent role for the caspase 3 activation in the hepatocellular apoptosis and fibrogenesis in NASH secondary to the methionine- and choline-deficient (MCD) diet model [21]. In addition, it was suggested that the high-cholesterol and high-cholate (HChCh) diet can induce intense atherogenic stimulus and additionally promote apoptosis through the oxidized low density lipoprotein (oxLDL). The oxLDL presents a proinflammatory and proapoptotic potential and can induce liver fibrosis [22].

Additionally, a new theory known as “lipotoxicity” has been focus of interest. Current evidences suggest that lipotoxicity represents the major mechanism of hepatocyte dysfunction leading to disease progression in NASH. According to this theory, lipotoxic injury occurs in the setting of the excessive traffic of free fat acids, especially saturated fatty acids (SFA), rather than due to simple steatosis. Probably, lipid accumulation occurs in parallel with the generation of lipotoxic metabolites, which are primarily responsible for the progression of liver disease [19, 23]. According to this theory, a high-carbohydrate and high-SFA diet contributes to the excessive circulation of free fat acids and promotes the development of insulin resistance. Moreover, it has been shown that lipotoxicity leads to cell injury and death, via apoptosis or necrosis, and may constitute an important proinflammatory and profibrogenic stimulus in chronic liver disease [19, 24].

Although the pathophysiology and diagnosis of NASH have been thoroughly studied, many investigators are still searching for a specific treatment. Several animal archetypes are indispensable for reproducing reliable models displaying characteristics as similar as possible to human NASH.

## 2. An Approach of Actual Experimental Models of NASH

Several animal models have been developed to represent the pathophysiology, morphological findings, biochemical changes, and clinical features of human NASH. Accordingly, the employed animals should present metabolic abnormalities such as obesity, insulin resistance, fasting hyperglycemia, dyslipidemia, and altered adipokine profile. Furthermore, an animal NASH model should display steatosis, intralobular inflammation, hepatocellular ballooning, perisinusoidal fibrosis, and susceptibility to liver tumors. However, the extrapolation of diverse animal models of NAFLD/NASH to the human disease may be limited by the difficulty in reproducing both the clinical and morphological conditions. The current models can be classified into genetic, nutritional, and a combination of genetic and nutritional factors. The following models are the most commonly used animal models of NAFLD/NASH development.

## 3. Genetic Models

To better understand the role of the specific genes involved in fatty liver formation, several studies have attempted to identify the deletion or overexpression of some genes that may be involved in the development of NAFLD/NASH. The genetic alterations can act diversely in various pathways but all culminate with lipid hepatocellular deposits. The main known genetic variation results in increased ingestion of calories, higher hepatic influx of fatty acids, neolipogenesis, and decreased hepatic oxidation of FFA and triglycerides [25]. However, the genetic models almost exclusively induce the biochemical alterations of NAFLD, and the addition of modified diets is frequently required in these models to induce the morphological changes found in human NASH [26].

### 3.1. SREBP-1c Transgenic Mice

In mammals, intracellular levels of cholesterol and fatty acids are controlled through a feedback regulatory system mediated by a family of transcription factors called sterol regulatory element-binding proteins (SREBPs). SREBP-1c transgenic mice overexpress the transcription factor SREBP-1c. In this model, the dysregulation of adipocyte differentiation leads to insulin resistance and diabetes. Similar to some forms of congenital lipodystrophies, the amount of systemic fat tissue is decreased, but the animals present significant hepatic lipid accumulation [27]. When SREBP-1c transgenic mice are fed a high-fat diet, pronounced hepatic steatosis can be induced in a few days. A standard diet is sufficient to induce steatosis, lobular and perivenular inflammation, and pericellular fibrosis in these animals after a period of 8 weeks [28, 29]. Therefore, the morphological findings are similar to many of the morphological findings observed in NASH. However, human NAFLD/NASH is frequently associated with metabolic syndrome and increased visceral fat, while this animal model develops lipid deposits exclusively in the liver. Consequently, despite the satisfactory pathological features, this model may differ from the clinical context of human NASH [30].

### 3.2. Ob/ob Mice

*Ob/ob *mice exhibit a spontaneous mutation in the leptin gene (leptin deficient). Leptin (from the Greek* lepthos *= thin) is a peptide hormone produced by adipose tissue. When this substance is secreted by adipocytes, it reaches the hypothalamus in the central nervous system and participates in the regulation of feeding behavior and energy bursts. Leptin promotes reduced food intake and increases energy metabolism by affecting the hypothalamic-pituitary axis and regulating neuroendocrine mechanisms. Leptin is also involved in the modulation of fibrogenesis and cell death [31–33].

**Figure 3:**
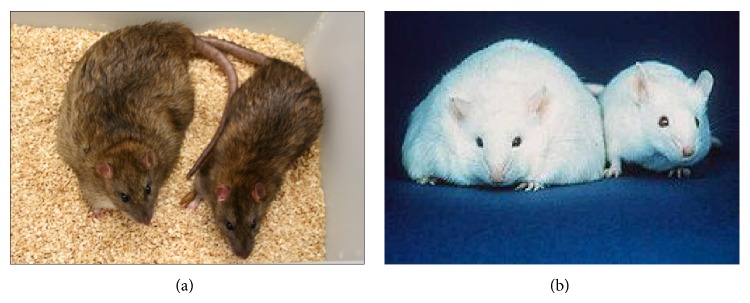
Genetic animal models used for the development of NAFLD/NASH: (a) Zucker rats—the recessive (*fa/fa*) genotype develops obesity and liver steatosis (at left side), while the dominant (*lean*) genotype is phenotypically normal (at right side of the picture); (b)* Ob/ob* mice—the mutant obese (*ob/ob*) mouse (at left side) may present threefold higher body weight relative to the wild type mouse (at right side of the picture) (The Jackson Laboratory).

In the* ob/ob *mice, a mutation in the leptin gene causes leptin deficiency and decreased interaction between leptin and its receptor; therefore, the* ob/ob *mice are hyperphagic, extremely obese, and inactive [26] (Figure 3). In addition, these animals have an altered metabolic profile and exhibit hyperglycemia, insulin resistance, hyperinsulinemia, and spontaneous development of fatty liver [34]. However, the utility of the* ob/ob *mouse model is limited by concerns with the development of liver fibrosis. In fact, the* ob/ob *mice are protected from fibrosis, and this phenomenon permitted the characterization of leptin as a key mediator of hepatic fibrogenesis. Because the progression to NASH does not occur spontaneously in this model, a secondary stimulus is necessary such as a MCD or high-fat diet or administration of lipopolysaccharide (endotoxin). Using these techniques, leptin-deficient mice can present reduced liver collagen deposits, which impairs the presence of important morphological characteristics of NASH such as fibrosis. Furthermore, mutations in the* ob *gene are not prevalent in obese subjects or patients with NASH, and leptin levels are not well correlated with the development of NAFLD/NASH [30, 35].

### 3.3. Db/db Mice

The* db/db *mouse model exhibits a spontaneous mutation in the leptin receptor gene (*Ob-Rb*). Although the* db/db *mice exhibit normal or elevated levels of leptin, they are resistant to the effects of leptin. Thus, the* db/db *animals are obese, present insulin resistance or diabetes, and develop macrovesicular hepatic steatosis. In addition, they may develop NASH after a second stimulus such as the intake of trans-fat or an MCD diet. When* db/db *mice are fed an MCD diet, they can develop significant liver fibrosis in contrast to* ob/ob *mice [36, 37]. The phenotype of the* db/db *mice better simulates the condition of human metabolic syndrome in many circumstances. However, these mice are limited because they do not spontaneously develop liver fibrosis or NASH without a second stimulus [30].

### 3.4. Zucker Rats

One of the most commonly used animal models of NAFLD, genetic obesity, and metabolic syndrome is the genetic model of obese Zucker rats (*fa/fa*). Zucker rats exhibit a spontaneous mutation in the leptin receptor (*fa *allele), which decreases the affinity of this receptor for leptin and changes the transduction signal. Zucker rats are homozygous for the* fa *allele, and heterozygous* fa *rats (*lean*) serve as the control. Zucker rats develop severe obesity and are hyperleptinemic, hyperphagic, inactive, obese, and insulin resistant (hyperinsulinemia, mild hyperglycemia, and hyperlipidemia) (Figure 3). Zucker rat hyperlipidemia is characterized by increased very low density lipoprotein (VLDL) and high density lipoprotein (HDL) without significant changes in low density lipoprotein (LDL) and reduced expression of the hepatic LDL-receptor [38]. In addition to significantly increased body weight, the Zucker rats depend on the heterozygote controls to reproduce because the metabolic abnormalities are also associated with high infertility rates in these animals [39].

In Zucker model, macro/microvesicular steatosis is diffusely present, mainly in the periportal area. No other sign of progression to NASH is detected. The Zucker rats also display low hepatic GSH and vitamin E levels and decreased catalase activity. As a result of leptin resistance, increased expression of SREBP-1c and carbohydrate response element-binding protein (ChREBP) may also be observed. The increased expression of SREBP-1c mRNA was accompanied by augmented levels of lipogenic enzymes and triglyceride accumulation in the liver [26].

Similar to* ob/ob *and* db/db *mice, Zucker rats do not present spontaneous development of NASH and require a second stimulus to induce the progression from steatosis to NASH. Accordingly, Zucker rats fed with a diet rich in saturated fat (60% of energy supply derived from lard) for 8 weeks developed severe micro/macrovesicular steatosis and progression to steatohepatitis. Liver injury was accompanied by increased levels of alanine aminotransferase (ALT), TNF-*α* and TGF-*β*, higher collagen deposition, and activation of hepatic stellate cells. Oxidative stress markers such as lipid peroxidation and protein carbonyl groups were increased, while the hepatic levels of reduced glutathione and antioxidant enzymes were diminished [40].

Although Zucker rats partially simulate human metabolic syndrome (obesity, insulin resistance, dyslipidemia, hyperinsulinemia, and liver), this animal model still has some disadvantages. Because leptin or leptin receptor mutations are rare in humans, Zucker rats may not reflect the clinical and pathological circumstances of the development of the NAFLD spectrum observed in humans. Furthermore, Zucker rats do not naturally develop steatohepatitis, are resistant to liver fibrosis, and require additional interventions to induce the progression of steatosis to NASH [26].

## 4. Nutritional Models

Nutritional models intend to mimic the bad alimentary habits that culminate in obesity and NASH. Some diets may cause consistent liver damage, steatosis, lobular inflammation, ballooning degeneration, and perivenular fibrosis similar to the histopathological findings of human NASH. The metabolic profile may also be changed, resulting in type II diabetes and high levels of triglycerides and cholesterol similar to the clinical characteristics observed in human NAFLD/NASH [26].

The mouse strain C57/BL6 is capable of simulating some human metabolic diseases and lipid disorders when submitted to different experimental models [41]. Similar to humans, adult C57/BL6 mice have a strong genetic tendency to develop obesity, hyperinsulinemia, and glucose intolerance, independently of the offered diet. This phenomenon may be further accentuated with the administration of a high-fat diet, which may result in obesity, insulin resistance, and steatosis similar to humans [32].

### 4.1. Methionine- and Choline-Deficient Diet

The MCD model is based on deficiency of methionine and choline, which are essential for liver *β*-oxidation and the production of VLDL. In addition to the methionine and choline deficiency, this diet contains a high quantity of sucrose (10% fat, 40% sucrose). The main results of the MCD diet are hepatocyte lipid accumulation and decreased synthesis of VLDL. Rats fed the MCD diet present weight loss (up to 40% at 10 weeks) and subsequent development of intense pericentral steatosis accompanied by necrosis and inflammation. The weight loss is based on reduction of corporal fat with a proportional decrease in liver size [32].

Mice fed the MCD diet also exhibit increased inflammatory responses through activation of liver macrophages due to the transcriptional factor nuclear factor kappa B (NF-*κ*B), which is an important modulator of inflammatory and cell survival responses. In addition to NF-*κ*B activation, concomitant augmentations in TNF-*α*, IL-6, and TGF-*β* levels are also observed [42]. The MCD diet also promotes higher expression of the intercellular adhesion-1 molecule (ICAM-1), vascular cell adhesion molecule-1 (VCAM-1), and macrophage chemotactic protein-1 (MCP-1), which results in increased activity, migration, adhesion, and accumulation of neutrophils and macrophages in the liver [43]. Although these proinflammatory mechanisms are similar to human NASH, and the MCD diet promotes real liver damage, the triglyceride and cholesterol levels are also reduced in contrast with obese patients with NAFLD/NASH. Other discrepancies in the metabolic profile of the MCD diet are reduced levels of insulin, glucose, and leptin, which are opposite to the effects of human NASH [44, 45]. Because the MCD diet is deficient in nutrients, it is not an ideal representative model of human NASH because the intrinsic nutritional factors observed in the human diet are not represented in the MCD diet.

### 4.2. High-Fat Diet

Because of the strong links between NAFLD and metabolic syndrome, animals have also been submitted to the “Western-style diet” model, which aims to induce obesity, insulin resistance, and liver damage. In these models, the animals are fed a high-fat diet in which 45–75% of the caloric intake is derived from fat and/or variations containing trans-fat or cholesterol. In the majority of the high-fat diet models, the degree of liver injury is not severe compared with the MCD model. However, the high-fat diet can represent the detrimental eating habits of the Western diet and mimic the etiology of NAFLD/NASH [25, 46].

A classic example of a high-fat diet model proposed by Lieber et al. (2004) is based on a liquid diet containing fat (71%), carbohydrates (11%), and protein (18%). The animals fed this diet presented biochemical alterations similar to the profile of NASH in humans. The rats also developed increased insulin levels, insulin resistance, hepatocellular lipid accumulation, oxidative stress, and TNF-*α* levels. However, the hepatic histopathology results displayed discrete signs of inflammation, mild steatosis, and the absence of the fibrosis progression in contrast with human NASH [47].

Other studies have intended to optimize the development of NASH through punctual modifications in the high-fat diet, such as enrichment with lard and cholesterol in a diet fed to Sprague-Dawley rats at 4, 8, 12, 24, 36, and 48 weeks. In this case, the liver weight increased at week 4, and hepatic steatosis was also observed. After week 8, the body weight started to increase. This finding was accompanied by augmented serum levels of FFA, cholesterol, and TNF-*α*. The serum ALT levels increased at week 12, and steatosis and inflammation occurred from weeks 12 through 48. Apparent hepatic perisinusoidal fibrosis did not occur until week 24, but collagen deposits were evident from weeks 36 to 48. This novel model may be potentially useful as a NASH model, although the main findings occurred only after week 24 [48].

Because of the presence of increased levels of ALT and triglycerides, liver neutrophil infiltration, and focal hepatocellular necrosis and apoptosis, high-fat diets are considered one of the best models to study the progression of steatosis to NASH. However, these models are limited because not all of the histopathological findings are consistent with those observed in human NASH, which is mainly due to the lack of progressive fibrosis. Furthermore, it takes a long time for the symptoms of the high-fat diet to significantly develop [25, 49].

Other techniques have been implemented to identify more severe histopathological alterations. In a study using C57/BL6 mice, an intragastric cannula was implanted to improve the intake of high-fat diets. In this model, an emulsion containing 37% of calories from fat (corn oil) and 39% of calories from dextrose was administered directly into the stomach. The treated mice developed hyperglycemia, hyperinsulinemia, hyperleptinemia, glucose intolerance, and insulin resistance. The mice also became obese with increased hepatic levels of fat. However, the histopathological analysis showed the development of mild hepatic steatosis [50]. In a study performed with Wistar rats using the same model of intragastric cannulation but with the administration of a trans-fat diet, the histopathological findings were more characteristic of the morphology found in human NASH [51].

Although this model can reproduce the histopathological pattern of NASH in humans, it failed to mimic the clinical and etiologic aspects because the administration of the diet to the animals was forced.

### 4.3. Atherogenic (High-Cholesterol and High-Cholate) Diet

Insulin resistance is a key event in the pathophysiological development of metabolic syndrome. Insulin resistance is associated with increased triglyceride and cholesterol levels and increased risk of cardiovascular disease. In some animal models, a relative insulin receptor insufficiency accompanied by increased plasma triglyceride concentrations during the development of obesity was observed, which is similar to the pathogenesis of insulin resistance in humans. Insulin resistant animals also exhibited an increased propensity to develop NAFLD/NASH [52, 53]. Based on these findings, other studies proposed new animal models fed an atherogenic diet with high levels of cholesterol and cholate, which could both lead to atherosclerosis and NASH. A mouse model showed that a high-cholesterol and high-cholate (HChCh) diet can induce varied degrees of liver inflammation and augmented collagen gene expression. The HChCh diet also stimulates the liver to produce TNF-*α*, which increases the inflammatory response and causes the progression of NASH [54].

Mice fed the HChCh diet presented progressive formation of hepatic steatosis, inflammation, and fibrosis after 6–24 weeks. The addition of 60% fat from cocoa butter in the HChCh diet accelerated the development of these histopathological alterations within 12 weeks. Furthermore, the fat-enriched HChCh diet induced higher oxidative stress and consequent increases in the activation of hepatic stellate cells and the expression of *α*-smooth muscle actin. These data suggest that the supplementation of HChCh diet with high-fat levels can cause hepatic morphological alterations of human NASH. However, metabolic status analysis of HChCh diet-fed animals showed an attenuation of the insulin resistance factors. In fact, during the course of the experiment, the animals lost 9% body weight, and the triglyceride levels were lower compared with the controls [25, 55].

Thus, although the HChCh diet enriched with fat can imitate the progression of human NASH, the metabolic profile showed important differences. Therefore, further studies are needed to assess whether changes in fat composition or addition of other dietary factors can improve the biochemical results of this model to increase its similarities to human NASH.

### 4.4. Cafeteria Diet

The incidence of metabolic syndrome, which is characterized by a combination of systemic dysfunctions including glucose intolerance, central obesity, dyslipidemia, and hypertension, has stimulated the development of new diet models mimicking the eating habits of the so-called modern Western diet. The aim of the cafeteria diet, also known as the Western diet, consists of industrially processed palatable foods containing high levels of fat, sugar, and salt. Therefore, this diet provides a robust model of human metabolic syndrome compared with the traditional high-fat diets and causes a phenotype of exaggerated obesity with glucose intolerance and inflammation [56]. However, this model presents some controversial results.

Young rats that received the cafeteria diet for 8 weeks developed metabolic syndrome along with obesity, higher hepatic weight, increased plasma levels of glucose, insulin and triglycerides, and insulin resistance [57]. In another study, hamsters fed the cafeteria diet for 15 weeks displayed significantly increased body weight and higher levels of plasma triglycerides, LDL-cholesterol, and glucose. Administration of the cafeteria diet resulted in a 35% decrease in adiponectinemia and insulinemia, augmented leptinemia, and increased homeostatic model assessment-insulin resistance (HOMA-IR). Although this diet induced insulin resistance and increased liver oxidative stress, proinflammatory mediators such as TNF-*α*, IL-6, and NF-*κ*B were not enhanced [58]. In accordance with these studies, the cafeteria diet is primarily a model of human metabolic syndrome.

Another study developed a variation of the cafeteria diet containing 65% fat (mostly saturated fat) administered to Wistar rats for 1 month. The animals presented consistent features of metabolic syndrome such as overweight, arterial hypertension, hypertriglyceridemia, hyperglycemia, insulin resistance, and liver steatosis but did not present significant liver inflammation or fibrosis [59]. In addition, Wistar rats fed standard chow with concurrently offered cafeteria food (cookies, cereals, cheese, processed meats, crackers, etc.)* ad libitum* for 15 weeks developed hyperphagia, resulting in severe obesity and prediabetes (glucose intolerance and hyperinsulinemia). This diet induced panlobular microvesicular steatosis, steatohepatitis, and chronic inflammation in white and brown adipose tissues [56]. Thus, according to these authors, this model closely reflects the etiopathogenesis of human NAFLD, although no study has demonstrated the presence of liver fibrosis.

### 4.5. Fructose

In humans, increased levels of fructose consumption, primarily in the form of corn syrup for soft drinks, are associated with increased severity of hepatic steatosis and fibrosis [60, 61]. Fructose, a monosaccharide primarily metabolized in the liver, controls the activity of glucokinase, which is the principle enzyme involved in hepatic glucose metabolism. Fructose is a potent and acute regulator of liver glucose uptake and glycogen synthesis. By interfering with glucose metabolism, the excessive fructose intake leads to postprandial hypertriglyceridemia, which increases visceral adipose deposition. Visceral adiposity contributes to hepatic triglyceride accumulation and insulin resistance by increasing the portal delivery of FFA to the liver [62–64].

Some animal and human studies show increased levels of triglycerides after ingestion of diets with fructose compared with diets containing complex carbohydrates or other sugars. This effect occurs due to increased lipogenesis in the detriment of gluconeogenesis, which results in increased hepatic synthesis of fatty acids and glycerol. The higher activity of lipogenic enzymes in the liver results in augmented levels of plasma total lipids and VLDL [63].

Mice submitted to an addition of 30% fructose in drinking water presented a fourfold increase in triglyceride levels and a marked increase in body weight along with steatosis after 8 weeks of ingestion [65]. No published data on animals demonstrated that administration of fructose alone can alter the metabolic parameters associated with NASH, but fructose has been reported to alter potent biochemical properties involved in promoting insulin resistance [66].

### 4.6. Association of Fructose with Other Nutritional Models

Dietary intervention using atherogenic or high-fat diets in addition to fructose represents a promising animal model for the induction of NASH with important similarities to the human disease.

Recently, a model diet was developed based on nutritional simulation of commonly consumed fast foods. In addition, the animals were maintained in conditions designed to promote sedentary behavior. These experimental circumstances are similar to those found in the majority of obesity patients in the Western culture. This archetype was termed the American lifestyle-induced obesity syndrome (ALIOS) model. The C57BL/6 mice were fed a high-fat diet containing trans-fats (partially hydrogenated vegetable oil) and significant amounts of fructose in corn syrup for 1 to 16 weeks. These animals presented obesity, hyperinsulinemia, and insulin resistance. The addition of high amounts of fructose in the diet increased the food intake and contributed to impaired insulin sensitivity. Moreover, the use of trans-fats induced hepatic lipid deposition and contributed substantially to hepatocellular injury. Although the ALIOS model led to severe steatosis, liver fibrosis was scarce [67].

Another study with mice showed that the administration of high-fat foods and high-fructose/sucrose liquids leads to a synergistic effect that may induce liver inflammation and fibrogenesis. Furthermore, the ingestion of sucrose along with fructose most likely accelerated the occurrence of hepatic macrovesicular steatosis and NASH [63].

Another type of combination diet is a high-fat, high-carbohydrate diet using medium-chain trans-fatty acids as high-fat component, and fructose and sucrose (55% and 45%, resp.) as high-carbohydrate nutrients. This diet caused a significant increase in hepatic triglyceride content, plasma ALT, and liver weight in mice after 16 weeks. Hepatic fibrosis, oxidative stress, hepatic collagen1 mRNA, and plasma cholesterol levels were also significantly increased. Mice fed a high-fat, high-carbohydrate diet can develop obesity and hepatic fibrosis. Moreover, these animals displayed a NASH-like phenotype and an important increase in hepatic ROS similar to human NASH [68].

In addition, the offering of a high-fat diet accompanied with fructose syrup for male and female mice resulted in some alterations of NAFLD/NASH after 16 weeks. These animals presented augmented ALT, triglycerides, IL-1*β* and TNF-*α* levels and insulin resistance and the histological alterations of NASH such as collagen deposition, macro/microvesicular steatosis, and liver fibrosis. Although these findings are consistent with NASH, in the female mice, hepatic inflammation and fibrosis were insipient [69].

The metabolic and histological effects of a diet based in the “fast food” composition were also evaluated. Mice were fed for 25 weeks with a “fast food” diet composed by 40% energy as fat (12% SFA, 2% cholesterol) or a high-fat diet composed by 60% energy as fat (1% SFA). Both diets were supplemented with high amounts of fructose. The high-fat diet resulted in obesity, insulin resistance, and steatosis, but inflammation was minimal, and there was not liver fibrosis. However, in mice fed the “fast food” diet, obesity and insulin resistance were also observed, but the liver histology showed steatohepatitis with pronounced hepatocellular ballooning and progressive fibrosis. The “fast food” diet also induced a gene expression associated with increased fibrosis, inflammation, endoplasmic reticulum stress, and lipoapoptosis. Thus, a “fast food”-based diet, composed by high saturated fat, high-cholesterol, and high-fructose, may simulate with elevated fidelity the features of the human metabolic syndrome and NASH [70].

## 5. Rabbit Models

Rabbits have also been utilized to the development of NASH. Rats have a very short prepubertal stage and they come into the adulthood in only one month. Therefore, rats are not the ideal animals to reflect the physiopathological state of the majority of children diseases. On the other hand, rabbits present around of 8 months of prepubertal stage and seem to be the factual animals for mimicking pediatric NASH. In this manner, it was created as a model to simulate pediatric NASH wherein young rabbits (4–6 weeks old) were fed with a high-fat diet (standard diet + 10% lard + 2% cholesterol) for 12 weeks. In this study occurred a generation of typical hepatic alterations of NASH, as liver steatosis, hepatocellular ballooning, severe portal inflammation (a major characteristic of pediatric NASH), perisinusoidal fibrosis, besides weight gain, augmented liver weight and higher levels of proinflammatory cytokines (TNF-*α* and IL-6), thereby producing the key features of pediatric NASH [71].

Another rabbit model was employed to simulate the human NASH. In this study, the animals were fed during 9 months with a diet supplemented with 0.75% cholesterol and 12% corn oil. After this long experimental period, the rabbits displayed increased serum and hepatic levels of total cholesterol. The livers presented a whitish and nodular aspect. In addition, hepatic gene expression for proinflammatory cytokines (TNF-*α*, IL-1*β*, IL-10, and IL-18) was significantly augmented. It was also observed significant increase of mRNA levels for TGF-*β* and collagen as well as advanced septal fibrosis. Moreover, the analysis of hepatic proteins and gene expression revealed an imbalance between antioxidant protection system and oxidative stress. Thus, this study showed a NASH model that features advanced fibrosis and may be useful for analyzing the molecular mechanisms of human NASH [72].

In another study, rabbits were fed with a high-fat diet supplemented with 20% corn oil and 1.25% (w/w) cholesterol for 8 weeks. In this model, the liver iron deposition derived from the augmented erythrocyte phagocytosis induced insulin resistance, hepatic lipid accumulation, Kupffer cell activation, mild fibrosis, and increased oxidative stress. These results revealed molecular mechanisms similar to those involved in the pathogenesis of human NASH [73].

## 6. NAFLD/NASH Models: A Schematic Summary

**Figure 4:**
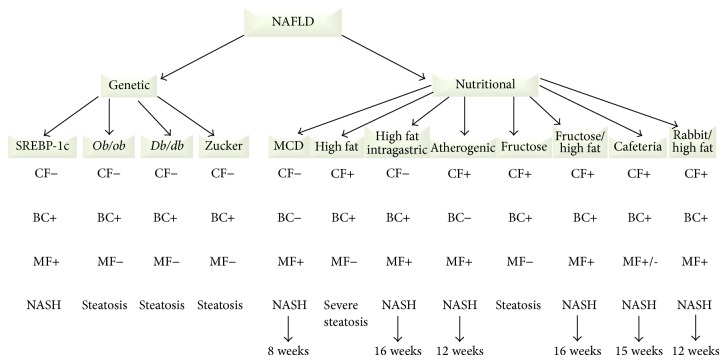
Comparison between the genetic (SREBP-1c,* Ob/ob*,* Db/db*, and Zucker) and nutritional (MCD, high-fat, high-fat intragastric, atherogenic, fructose, fructose/others, cafeteria, and rabbit high-fat diets) animal models concerning to the main clinical features (CF), biochemical changes (BC), morphological findings (MF), and the occurrence of liver steatosis or NASH.

Figure 4 intends to schematically present an analysis of the main morphological findings (MF), biochemical changes (BC), and clinical features (CF) found in the different animal models of NAFLD/NASH.

The SREBP-1c mutant mice fed a standard diet exhibited steatosis, liver inflammation, and fibrosis, which includes many of the morphological findings observed in NASH (MF+). Similar to humans, these animals develop insulin resistance and diabetes (BC+). Human NASH is frequently associated with increased visceral fat, and these animals present an exclusive lipid deposit in the liver. Because this mouse models included a genetic modification, the clinical features differ from the human conditions (CF−). Therefore, this model is incomplete if the full context of human NASH needs to be considered.

The* ob/ob *and* db/db *mutant mice are also genetic models. Although these mice were extremely obese and inactive, their clinical features are incompatible with the features of human disease because the etiology of NAFLD/NASH is not genetic in the vast majority of the cases (CF−). These animals have an altered metabolic profile, exhibiting hyperglycemia, insulin resistance, and hyperinsulinemia (BC+). Although these animals spontaneously develop steatosis, progression to NASH does not occur without additional secondary stimuli mainly using special diets (MF−).

Zucker rats are a genetic model of obesity and metabolic syndrome. Because leptin or leptin receptor mutations are rare in humans, Zucker rats do not reflect the clinical conditions of NASH observed in humans (CF−). These rats partially simulate human metabolic syndrome (obesity, insulin resistance, dyslipidemia, and hyperinsulinemia) (BC+). Zucker rats have been one of the most commonly used models of NAFLD and present diffuse steatosis mainly in the periportal area. However, a second stimulus is required to induce the progression of steatosis to NASH (MF−).

The MCD diet model results in many histological abnormalities that are similar to human NASH (MF+), but this model is not associated with insulin resistance or other biochemical alterations (BC−). MCD diet-treated rodents typically present decreased body weight. Furthermore, this model does not reflect human dietary habits (CF−).

The high-fat diet represents a realistic example of the modern Western lifestyle, and rodents treated with this diet frequently become obese (CF+). Although these animals show insulin resistance and increased triglycerides and VLDL levels resembling the profile of human NASH (BC+), the histopathological findings display steatosis without liver fibrosis (MF−). Thus, this model fails to simulate the progression to NASH.

The high-fat diet with intragastric cannula model leads to hyperglycemia, hyperinsulinemia, hyperleptinemia, and insulin resistance (BC+). The histological alterations show severe steatosis with progression to fibrosis (MF+) similar to human NASH. However, this model does not mimic the clinical and etiologic factors of NAFLD/NASH because the diet is forcibly administered (CF−).

The atherogenic diet, which includes high levels of cholesterol and cholate, accurately simulates the etiologic and clinical factors observed in human NASH (CF+). The rodents fed this diet present progressive hepatic steatosis, inflammation, and fibrosis after 12–24 weeks of induction, which replicate the histopathological findings of human NASH (MF+). However, the metabolic status is not consistent with human NASH because of the presence of insulin resistance attenuation, decreased body weight, and lower triglyceride levels (BC−).

The cafeteria diet exhibits high similarity with human dietary habits, and rodents fed this diet exhibit obesity, higher visceral adiposity, and augmented liver weight (CF+). In addition, insulin resistance and increased plasma levels of glucose, insulin, and triglycerides resemble human NASH (BC+). However, the histopathological findings may display steatosis with or without inflammation, but there is no liver fibrosis. As a consequence, this model simulates partially the progression to NASH (MF+/−).

In the fructose diet models, clinical features can simulate the etiology of human NASH (CF+). Some studies show increased triglyceride levels, with potential induction of insulin resistance (BC+). However, no published animal data demonstrated that administration of fructose alone can alter the morphological findings and progression to NASH (MF−).

Nutritional models using combinations of high-fructose and other high-fat diets cause clinical and etiology characteristics similar to human NASH (CF+). Biochemical parameters such as insulin resistance and augmented triglycerides levels were also observed (BC+). The histopathological findings, such as severe steatosis, inflammation, and progression of hepatic fibrosis, were also observed after 16 weeks of diet administration (MF+).

Nutritional models applied in rabbits are associated with the etiologic characteristics that are similar to human NASH (CF+), once the animals had been fed with a high-fat diet. Biochemical parameters such as increased serum levels of total cholesterol and triglyceride were also observed (BC+). The histopathological findings as severe steatosis, inflammatory infiltration, hepatocellular ballooning, severe portal inflammation, and septal fibrosis were also found after 16 weeks of diet administration (MF+).

## 7. Conclusion

The presentation of the genetic and nutritional models and combinations of these models confirms the difficulties in identifying an accurate model of human NASH in rodents. Some models produce consistent morphological findings, but their induction differs significantly from the pathophysiology of human NASH. Other models accurately represent the clinical and etiological contexts of this disease but do not simulate the histopathological observations involving the progression from simple steatosis to liver fibrosis. Few rodent models produce effects that simulate human NASH, but these effects may occur after a long time. Rabbit models can produce consistent morphological findings, with characteristics very similar to the pathophysiology of human NASH, including advanced fibrosis.
